# Performance Monitoring, Subordinate’s Felt Trust and Ambidextrous Behavior; Toward a Conceptual Research Framework

**DOI:** 10.3389/fpsyg.2022.758123

**Published:** 2022-05-03

**Authors:** Farooque Ahmed, Shuaib Ahmed Soomro, Fayaz Hussai Tunio, Yi Ding, Naveed Akhtar Qureshi

**Affiliations:** ^1^Sukkur IBA University, Sukkur, Pakistan; ^2^Shaheed Zulfiqar Ali Bhutto University of Law, Karachi, Pakistan; ^3^Central University of Finance and Economics, Beijing, China

**Keywords:** performance, monitoring, trust, leader-member exchange (LMX), ambidextrous behavior, ambidextrous leadership

## Abstract

The present research proposes an electronic performance monitoring framework based on ambidextrous leadership and social exchange theories in a dynamic environment. It reviews and integrates essential literature on electronic performance management (EPM), trust, and ambidextrous behavior. For this, authors have reviewed relevant literature on various themes and underpinned them for managing EPM. The study emphasizes individuals’ psychological foundations that demonstrate trust behavior and relationship with their leaders. Eventually, through an ambidextrous approach, managers gain steady performance and relationships with their subordinates through EPM. The study shows that ambidexterity benefits organizations; it enhances employees’ resources, resulting in enhanced performance that leads to the performance of an organization. The authors discuss the theoretical as well as practical implications of this study.

## Introduction

Leader’s role has always remained in the limelight in business and academia (Cortellazzo et al., 2019). In this era of quick change, evolution, and technological improvements, organizational leaders expect their subordinates to be experts in dealing with current and upcoming challenges (Hunter and Perreault, 2007). The literature on leadership describes that employees and associates want humble, insightful, empowering leaders (Owens et al., 2013). Contrary to the literature, business leaders in practice exercise swashbuckling in all-seeing and all-doing ways in an organization to get work done. Hence, these leaders are not humble and quiet (Johnson et al., 2012), they are the exact opposite of what we have in literature and what practicing leaders do in business (Matos et al., 2018). These swashbuckling practices create a dilemma for leaders. They think about what kind of leadership style they can apply to succeed in the competitive market that exercises a humble way to get their work done. At the same time, the correct type of leadership depends on the business situation. For instance, competition among service providers requires leaders to focus on service employees’ quality service to meet increasing customer demands (Agnihotri et al., 2017). Leaders who combine service quality with sales enable competitive advantage which helps them to motivate workers to perform simultaneously (Gabler et al., 2017).

In today’s competitive environment, sales leaders have doubted their subordinates’ performance working in the field. As a practice, they cannot be with them working in the area, so they pay surprise visits to see how they are workings in the field. The sales leaders’ objective is to meet the service quality and sales results through their subordinates. Typically, sales employees work remotely and away from their leaders (Cascio, 2000). Physical space separates them and may affect their relationship, leading to a decline in their performance (Wieseke et al., 2008). In contrast, technological gadgets provided leaders with the convenience of accessing their subordinates’ performance in the field. In this regard, they can assess and manage their performance efficiently to achieve subordinates’ success. With the arrival of electronic performance monitoring (EPM), leaders are further benefiting from a myriad of valuable services like performance measurement and improvement, productivity reports and communication services.

Prior research has shown employees’ significant concerns with monitoring; consequently, it creates a working environment by reducing trust and unpleasant working relationships (Greengard, 1996; Lewis and Sobhan, 1999). It has adverse outcomes like work stress (Kolb and Aiello, 1996) and perceived distrust (Frey, 1993; Ariss, 2002; Smith and Tabak, 2009). Accordingly, organizations demonstrate ambidextrous behavior among leaders to handle subordinate behavior (Kao and Chen, 2016). Such behavior labels employees simultaneously to exploring new skills and exploiting present skills in their job responsibilities and obligations (Mom et al., 2009; Kauppila and Tempelaar, 2016). Few firms, for example, train sales staff to simultaneously engage in cross-selling and up-selling (Jasmand et al., 2012; Johnson and Friend, 2015). Previous studies described how employees and organizational performance are certainly affected by leaders’ ambidextrous behavior (Auh and Menguc, 2005; Cao et al., 2009; Mom et al., 2009; Kauppila, 2010; Patel et al., 2013). More precisely, individual-level ambidexterity has been found to increase sales performance (Jasmand et al., 2012).

Previous research shows that ambidexterity benefits many companies, it may also enhance employees’ resources, resulting in greater performance (Gabler et al., 2017). There is progress in exploring individual ambidexterity and its influence on workers (Kao and Chen, 2016; Kauppila and Tempelaar, 2016; Gabler et al., 2017). More is needed to explore how sales leaders support subordinates’ ambidextrous behavior. Besides this, subordinates expect trust and respect from their leaders (Ullrich et al., 2009); they want independence and dignity in their employment (Deci and Ryan, 2002). Research has shown if EPM employees think that they are being viewed with integrity and dignity by their leader, they can respond by believing the leader more (McNall and Roch, 2009). The positive effect led to leader-member exchange (LMX) due to a rise in the trust level (Newcombe and Ashkanasy, 2002). Further, individuals who experience high-quality LMX openly address challenges in achieving their job goals amid the monitoring process (Audenaert et al., 2019). The ambidextrous individuals refine and update their expertise, knowledge, and skills (Schnellbächer and Heidenreich, 2020), specifically in a sales job to build and retain clients. In this track, EPM purpose can enhance an employee’s desire to progress in knowledge, skills, and abilities (Ravid et al., 2020).

This paper contributes to the existing literature in many ways.

• First, in light of ambidextrous behavior (Gabler et al., 2017), and a future research call from Ravid et al. (2020), there is a need to study EPM to achieve employees’ engagement in exploitation and exploration behaviors.
• Second, past research on the developmental purpose of EPM was restricted to attitudinal outcomes (Wells et al., 2007). We add to the literature through its behavioral effects on LMX and ambidextrous behavior. Hence, understanding of the perceived purpose of EPM will be enhanced.
• Third, to better record reciprocal reactions of subordinates after their perception of EPM as developmental, the research proposed serial mediation of felt trust and perceived LMX quality. The serial mediation can help in gaining a solid knowledge of underlying mechanism between perceived developmental EPM and ambidextrous behaviors.
• Fourth, the study is proposed in sales field where subordinates normally work away from their leader (Cascio, 2000). Under fixed EPM, sales people can only achieve sales goals consistently by demonstrating in ambidextrous behavior. Keeping monitoring as developmental, this model offers trust, respect and LMX to subordinates with purpose to reciprocate in the form of behavioral ambidexterity.
• Finally, the research framework contributes to social exchange theory (Blau, 1964) that posits that how individual engage in exchange relationship that explicitly brings a win– win situation for all the stakeholders.

## Literature Review

### Electronic Performance Monitoring and Its Developmental Purpose

Electronic performance monitoring (EPM) is an integral part of new information mechanisms and working environments. Monitoring employees’ performance helps companies determine whether to pay or not (Alder, 2001). Motivations for EPM implementation are to assess both constructive (productivity, task performance) and detrimental behaviors of employees, like counterproductive work behaviors (CWBs) (Tomczak et al., 2018). EPM can enhance employees’ performance (Bhave, 2014), and it has shown its positives and negatives like data protection, health monitoring, and safety protection (Alge and Hansen, 2014), stress (Kolb and Aiello, 1996), and distrust (Frey, 1993; Ariss, 2002; Smith and Tabak, 2009).

Further, organizing constructs relevant to monitoring indicate that perceivable monitoring features influence the employees’ feelings, opinions, and assessments about monitoring and then the effect on thoughts such as fairness, trust, and happiness (Stanton and Weiss, 2000). Purpose, probably more than any other EPM feature, can most clearly express what a company values and anticipates from employees (Wells et al., 2007; Jeske and Kapasi, 2018). When observed as developmental, monitoring is regarded as fairer compared to when it is alleged as a warning to future conduct (Wells et al., 2007). Expanding on the social exchange model of McNall and Roch (2009) on EPM reactions, we believe that the perceived developmental purpose of EPM can be a base for felt trust and perceived LMX quality and ultimately ambidextrous behavior of sales workers.

### Felt Trust

The felt trust refers to a judgment about the degree to which others trust you (Gill et al., 2019). To feel others’ trust, the individual has to recognize that the trustor has the impression that the trustee will complete specific actions worthy of the trustor (Lau et al., 2007). Subordinates who sense trust realize that another party expects intelligent behavior from them without monitoring (Lau et al., 2014). The subordinate’s felt trust has a significant positive impact on subordinate’s psychological empowerment (Karunarathne, 2019). In the context of leader and subordinate relationships, trust can lead to subordinates’ positive behavior toward the leader (Dirks and Ferrin, 2002) and brings exchange relationship quality and teamwork (Chiu and Chiang, 2019). If trusted partnerships are not formed and sustained, salespeople and sales managers alike can waste precious time on efforts intended to defend themselves from each other (Strutton et al., 1993). Since workers cannot know instantly to what degree their leader trusts them, the sense of trust is likely to evolve based on behavioral and situational signals perceived as demonstrations of trust or absence thereof. Therefore, in our suggested framework based on Social Exchange theory (Blau, 1964), we place the felt trust after the perceived developmental purpose of EPM and before the perceived LMX. Accordingly, subordinates can feel the trust of their supervisors if they perceive the EPM’s purpose as developmental. Further, the felt trust can increase their perception of LMX quality and obligation to pay back as social exchange.

### Perceived Leader-Member Exchange

In the exchange relationship, when an individual feels the delight of receiving more support than allocated, he/she considers it as a high-quality relationship (Byun et al., 2017). Consequently, a high-quality LMX relationship evolves with a high degree of loyalty and mutual trust between a leader and his members (Sparrowe and Liden, 2005). The higher the perceived quality of the LMX, the more inspired members are to participate in the social exchange with the leader to keep gaining tangible benefits, e.g., information, and intangible benefits, e.g., the leader’s trust (Erdogan and Enders, 2007). The high-quality LMX gives rise to employees’ psychological empowerment (Rafique et al., 2022). Researchers have agreed that to respond to high-quality LMX, members will go beyond the necessary in-role performance and participate in organizational citizenship behavior to maintain a stable social exchange (Ilies et al., 2007). Employees receiving preferential treatment from superiors should promote positive actions, e.g., ambidextrous behavior (Rhoades and Eisenberger, 2002).

### Ambidextrous Behavior

Ambidextrous behavior is the tool of employees to effectively adapt to complex scenarios by effectively controlling their exploitation and exploration responses. In the organizational setting, individual exploitation is the ability to maintain concentration on the relevant content and the task at stake, whereas exploration includes the quest for innovation and creativity (Good and Michel, 2013). Findings reveal that such an ambidextrous technique contributes to more consistency in their efficiency by adding to their competitive advantage. When individuals are skilled in two qualities, they can become adaptable, happy to extend their perceptions to possibilities, and function well according to situation (Zhang et al., 2019). Considering the worth of ambidextrous behavior, research constantly redefines it as different conflicting demands like adaptability versus alignment (Gibson and Birkinshaw, 2004), flexibility versus efficiency (Adler et al., 1999; Yu et al., 2020) creativity versus attention to detail (Sok and O’Cass, 2015), sales and service quality (Agnihotri et al., 2017). Specifically for sales workers, it is central to behave ambidextrously (Van der Borgh et al., 2017). For example, they can achieve sales growth by selling higher quantities to current customers through exploitation and prospecting new clients to achieve sales growth through exploration. Hence, we have kept ambidextrous behavior as an outcome in a social exchange process to create a win–win situation.

## Theoretical Framework and Propositions Development

### Developmental Purpose of Electronic Performance Monitoring and Subordinate’s Felt Trust

Subordinates cannot know immediately to what degree their leader trusts them. Feelings of being trusted or not trusted are expected to occur after perception of behavioral and situational signals as demonstrations of trust or absence thereof (Lau et al., 2014). Normally, monitoring is a signal of no confidence, and it is expected to be perceived by subordinates as a symbol of distrust (Frey, 1993; Ariss, 2002; Smith and Tabak, 2009). However, the employee’s perception that the intent of EPM is developmental would give the impression that the individual is capable of the time and investment needed for development efforts. The perception of EPM as developmental could convey the signal to the individual that you are trusted and respected (Wells et al., 2007). Therefore, it is expected that the perceived developmental purpose of EPM can induce feelings of being trusted in subordinates. Thus, we propose that;

P:1 There will be a positive relationship between perceived developmental EPM and felt trust.

### Developmental Electronic Performance Monitoring and Leader-Member Exchange

Earlier research has criticized EPM as it invades privacy, increases stress, decreases job satisfaction, and creates a work environment characterized by weakened trust and undesirable work relationships (Piturro, 1989; Greengard, 1996; Lewis, 1999). The belief that the function of EPM system is to limit employees from practicing unnecessary and undesirable behaviors may indicate that the company has neither confidence nor trust. And employees can’t work satisfactorily in the absence of monitoring. Such a perception would not probably convey an acknowledgment as a respected member and instead may consider the employee for investigation (Wells et al., 2007). So, in the exchange relationship, it is evident that the deterrent perception of electronic performance monitoring can harm the relationship between a subordinate and his/her leader. On the other hand, this relationship can be improved when subordinates perceive the purpose of monitoring as developmental. When EPM employees think that they are being viewed with integrity and dignity by their leader, they can respond by believing the leader more (McNall and Roch, 2009). The developmental motive for EPM (Tomczak et al., 2018) may positively impact the perception of relationship quality among subordinates. Hence, we expect that the perceived developmental purpose of EPM can enhance the perception of subordinate’s LMX quality. Thus, it is suggested that

P:2 There will be a positive relation between developmental EPM perception and subordinate’s perceived LMX.

### Developmental Electronic Performance Monitoring and Ambidextrous Behavior

It is well established in organizational literature that employees’ attitudes and behaviors are linked to their electronic performance monitoring (Tomczak et al., 2018). So, the way EPM is introduced and conveyed to workers becomes critical. The broad difference in views on EPM indicates that subordinates don’t respond similarly to monitoring in all situations (Alder, 2001). The findings has shown that when EPM is seen as developmental, it is acknowledged as fair and brings a commitment to the organization and felt obligation (Wells et al., 2007). In the organizational setting, individual exploitation is the ability to maintain concentration on relevant content and the task at stake, whereas exploration includes’ quest for innovation and creativity. However, employees can succeed in ambidexterity by situational alignment (Gibson and Birkinshaw, 2004).

In contrast, organizational structures are occasionally needed to facilitate behavioral ambidexterity individually (Volery et al., 2015). They proposed developing ambidexterity through a suitable organizational framework, comprising attributes of support, discipline, stretch, and trust (Gibson and Birkinshaw, 2004). They demonstrate that mutual respect, transparency, and trust among personnel lead to promoting an environment of information sharing that has a meaningful impact on individual ambidexterity (Ajayi et al., 2017). Recently, Ravid et al. (2020) expected that the developmental purpose of EPM could enhance an employee’s desire to progress in existing skill or develop a new one. Therefore, we have supposed our third proposition.

P:3 There will be a positive relation between developmental EPM perception and Ambidextrous behavior.

### Felt Trust and Perceived Leader-Member Exchange Quality

Subordinates significantly like trust and respect from their leader and organization (Ullrich et al., 2009). Felt trust relates to subordinates’ opinions about how strongly their superiors trust them (Lester and Brower, 2003). From a social exchange point of view, this indicates their leader’s willingness to spend additional effort to strengthen and enhance their relationships, which excites subordinates to contribute to the social exchange (Dulebohn et al., 2012). While few scholars have examined the effect of felt trust on quality LMX, results were either positive (Lau et al., 2014; Kim et al., 2018) or negative (Baer et al., 2015). However, without trust, it is unlikely to have high-quality LMX (McKnight et al., 1998), as trust enables a more efficient exchange partnership between two parties (Colquitt et al., 2007). Therefore, it would be beneficial to add this relationship in the context of the sales force. Hence, we propose,

P:4 There will be a positive relationship between subordinate’s felt trust and perceived LMX quality.

### Felt Trust and Ambidextrous Behavior

Being under the umbrella of trust can lead to a sense of responsibility or obligation in trusted individuals to perform tasks or roles required by trustors (Lau et al., 2014). The social exchange is one mechanism through which trust can bring positive work results (Gill et al., 2019). Earlier work on felt trust has shown its importance for multiple positive organizational and employees’ outcomes like job satisfaction, less intention to leave, organizational citizenship, job performance, psychological empowerment, and trust in the supervisor (Lester and Brower, 2003; Brower et al., 2009; Gill et al., 2019). If subordinates recognize that their leader trusts them, their organizational self-esteem is improved, encouraging them to perform much better in the field (Lau et al., 2014). Research has also shown a positive link between trust and an individual’s behavior to provide novel ideas (Rodrigues and Veloso, 2013), an employee’s intrinsic motivation and experience of mastery (Bernstrøm and Svare, 2017). Trust as an organizational factor also encourages ambidexterity at an individual level (Zhang et al., 2019). Therefore, we expect that a subordinate will demonstrate ambidextrous behavior if he feels his leader’s trust. Thus, we propose

P:5 There will be a positive relationship between felt trust and ambidextrous behavior.

### Felt Trust as a Mediator Between Perceived Developmental Electronic Performance Monitoring and Leader-Member Exchange Quality

The positive perceptions toward leaders are important in developing high-quality LMX since these offer pleasant feelings to subordinates and direct subordinates to have faith in continuous advantage from the exchange relationship (Liao and Chun, 2016). Developing subordinates’ competencies through EPM can enhance LMX quality as it leads to the positive perception of subordinates toward their leader. This perception could convey the message to the subordinates that they are trusted (Wells et al., 2007). The degree to which a manager trusts a subordinate has implications for the nature of the relationship between the subordinate and the supervisor and for autonomy at work (Seppälä et al., 2011). Trust is also recognized as a significant mediator between different organizational activities and worker outcomes (Bernstrøm and Svare, 2017). Felt trust has also gained considerable support in the existing literature as a mediating variable. Earlier, Falk and Kosfeld (2006) tested the mediating effect of felt trust between monitoring and intrinsic motivation, contributing to a decrease in trust. In other study, the relationship between monitoring and intrinsic motivation and monitoring and mastery was fully mediated by the felt trust (Bernstrøm and Svare, 2017). Therefore, this research proposes,

P:6 Felt trust will mediate the relationship between perceived developmental EPM and perceived LMX quality.

### Perceived Leader-Member Exchange and Ambidextrous Behavior

Subordinates with a high LMX partnership believe that they are operating in an inspiring psychological atmosphere. Obligatory, they participate in discretionary processes and innovative work by responding positively to their leader’s favors (Atwater and Carmeli, 2009; Volmer et al., 2012). Subordinates in high-quality LMX partnerships are considered to be knowledgeable and credible, and acquire additional tools relevant to tasks and relational help to execute assignments (Gu et al., 2015) efficiently. Earlier research revealed that high-quality relationships between a leader and members create a psychological atmosphere that promotes salespeople’s empowerment by raising subordinates’ feelings of autonomy and care (Martin and Bush, 2006). Research has confirmed the subordinates’ empowerment through leadership style brought service-sales ambidexterity (Yu et al., 2013). In the near past, cross-functional teamwork, association, and confidence with the supervisor were the features that visibly led to behavioral ambidexterity of subordinates (Yu et al., 2013; Patterson et al., 2014; Van der Borgh et al., 2017). Therefore, we argue that the quality of LMX can influence the ambidextrous behavior of sales employees. Thus, we propose that,

P:7 There will be a positive relationship between perceived LMX and ambidextrous behavior.

### Perceived Leader-Member Exchange as a Mediator Between Felt Trust and Ambidextrous Behavior

Felt trust increases followers’ beliefs in their functioning capacity, leading to success in tasks and different behaviors (Zheng et al., 2019). From the social exchange view, members who are trusted feel obligated to keep that trust and reciprocate by working hard to enhance their task performance (Lau and Liden, 2008). The output of LMX represents a particular type of social exchange within the company (Cropanzano et al., 2002) that could be a possible mediator because of psychological impact that a leader exerts. According to the social exchange theory, leaders’ positive actions may build liabilities among subordinates by creating a favor exchange. The favors exchange leads subordinates to feel advantages at many levels, including organizational resource control, competence, consideration, and trust (Li et al., 2012). In a recent study, perceived LMX was found as a mediator between the leader’s trust and subordinate’s task performance (Byun et al., 2017). So, it is expected that perceived LMX can mediate the relationship between the perceived developmental purpose of EPM and ambidextrous behavior. Drawing on the social exchange theory, we propose

P:8 Perceived LMX by subordinate will mediate the relation between felt trust and ambidextrous behavior.

### Felt Trust and Leader-Member Exchange as Mediators Between Perceived Developmental Electronic Performance Monitoring and Ambidextrous Behavior

**FIGURE 1:**
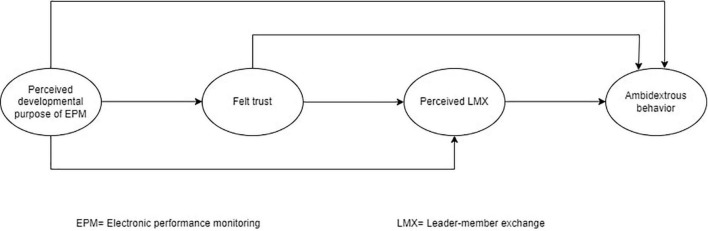
Research model.

In this research model as revealed in Figure 1, developmental EPM offers a signal of being trusted to subordinates, which leads to the perception of high LMX and consequently enables them to engage in exploitation and exploration in the form of ambidextrous behavior. Reciprocity is the social exchange law whereby the two sides satisfy their gains and accomplish exchange (Cropanzano and Mitchell, 2005). Social exchange relationships also have implicit, instead of explicit, obligations regarding reciprocity. Trust between two people is imperative to continue the relationship (Blau, 1964). Subordinates are more willing to recognize a leader’s authority if they believe they are trusted by the leader (Zheng et al., 2019). Earlier research has recommended that employees who are assumed competent are inclined to construct and sustain a higher LMX level with their superiors, but those who are considered ineffective are expected to maintain a lower LMX quality (Graen and Uhl-Bien, 1995; Gerstner and Day, 1997; Liden et al., 1997). Subordinates show more desirable habits, like higher performance, when the standard of LMX is higher rather than poor, since they want to give back the advantages of their supervisor’s high-quality relationship (Gerstner and Day, 1997). Against these characteristics of Felt trust and LMX, we expect that felt trust and LMX will mediate the relationship between perceived developmental EPM and ambidextrous behavior. Drawing on social exchange theory (Blau, 1964), we propose,

P:9 Felt trust and perceived LMX will mediate the relationship between Perceived developmental EPM and ambidextrous behavior.

## Conclusion

In this competitive world, organizational leaders want their workers to be capable of dealing with current and future problems on the job (Hunter and Perreault, 2007). In contrast to popular belief, most business leaders in practice are swashbuckling, strong, in all-doing and in all-seeing. These are not rulers who are polite and calm (Johnson et al., 2012). This is completely opposite to what we read in the literature and what they do (Matos et al., 2018).

The arrival of EPM has added more power to leaders as they can monitor their subordinates continuously. However, a major monitoring issue is that it produces working environments marked by reduced trust and unpleasant working relationships (Greengard, 1996; Lewis, 1999). We believe that reduced trust and unfriendly relationships can distract the performance of subordinates working in the field. Hence, here is a solid case for monitoring as developmental instead of a deterrent. It is the starting point of our social exchange framework. Thus, we propose that if subordinates perceive EPM as developmental, they can feel their sales manager’s trust and the perceived quality of LMX. According to social exchange theory (Blau, 1964), leaders’ positive actions may build liabilities among subordinates by creating a favor exchange.

Consequently, we have reasoned that subordinates’ ambidextrous behavior can be an outcome of the perceived developmental purpose of monitoring. Our framework is consistent with the social exchange model of McNall and Roch (2009) on reactions of employees to the developmental purpose of EPM.

In the context of the leader-subordinate relationship, trust can lead to subordinates’ positive behavior toward the leader (Dirks and Ferrin, 2002) and brings exchange relationship quality and teamwork (Chiu and Chiang, 2019). Being under the trust can lead to the sense of responsibility or obligation in trusted individuals to perform tasks or roles as required by trustors (Lau et al., 2014). Accordingly, we have suggested a positive relationship among felt trust, perceived LMX, and ambidextrous behavior. The entire relationship between variables is backed by social exchange theory (Blau, 1964). Hence, this inclusive framework can bring mutual gain for a sales leader and sales subordinates working in the field. Under the umbrella of trust and LMX, sales workers are expected to demonstrate ambidextrous behavior if they perceive monitoring as developmental. Sales Managers will also gain steady performance and professional partnership with their subordinates by creating a trustful environment. In this framework, we have well-adjusted the power between leader and associate and termed it as a win-win situation for all.

We contribute to social exchange theory by providing a novel and useful framework in the context of sales. Based on reciprocity, this analytical framework demonstrates a positive relationship between the perceived developmental purpose of EPM, Felt trust, perceived LMX quality, and ambidextrous behavior of sales workers. Earlier studies on the developmental purpose of EPM were limited to attitudinal outcomes (Wells et al., 2007). We add to the literature through its behavioral effects on LMX and ambidextrous behavior. Hence, understanding of the perceived purpose of EPM will be enhanced. In earlier studies, the relationship between felt trust and leader- member exchange was conflicting. Few studies exposed it as positive (Lau et al., 2014; Kim et al., 2018) and others revealed it as negative (Baer et al., 2015). But, we believe that in the context of sales, the relationship will be positive. Previous research has shown creativity, and efficient execution of assignments as positive outcomes of LMX; (Gu et al., 2015). Thus, ambidextrous behavior is expected as the positive outcome of perceived high-quality LMX, which is another contribution to literature. We expect this entire framework as a win-win situation for sales leaders and subordinates and will find the right place in literature.

### Practical Implications

Sales managers in electronic performance monitoring need to value subordinates’ esteem. There may be some organizational factors that potentially encourage managers to monitor employees. However, the purpose should be developmental instead of deferral. There are many logics for this. First, it is the ethically right thing to do. Second, evidence from many case studies shows that subordinates monitored through EPM feel additional stress than associates observed through other methods (Kolb and Aiello, 1996). Third, the expected benefits of monitoring may be reduced or even removed if workers have an adverse reaction to the EPM system (Jeske and Santuzzi, 2015). It sheds light on the value of trust in the relationship. Since workers cannot know instantly to what degree their leader trusts them, the sense of trust is likely to evolve based on behavioral and situational signals perceived as demonstrations of trust or absence thereof (Kim et al., 2018). Hence, through perceived developmental EPM, subordinates will feel the trust of their leader. Felt trust is specifically essential for sales workers as they work remotely and physically away from their leader. This physical distance may decrease belonging between sales leaders and salespeople (Cascio, 2000). However, trust is recognized as the single most critical feature of any successful professional partnership (Kramer, 1999). Therefore, leaders will be able to enjoy a required professional collaboration with their subordinates. Subordinates will also perceive high LMX relationship quality on the perception of developmental EPM and felt trust.

Further, felt trust has importance for multiple positive organizational and employees’ outcomes like job satisfaction, less intention to leave, organizational citizenship, job performance, psychological empowerment, and trust in the supervisor (Lester and Brower, 2003; Brower et al., 2009; Gill et al., 2019). Salespeople serve a pivotal role in successfully implementing the organizational strategy of selling new and existing products (Van der Borgh et al., 2017). In this framework, the most important implications for sales managers can be for ambidextrous behavior of salesforce. If they want subordinates to sell new and existing products, they can achieve it by promoting developmental EPM and exchange relationship quality.

### Limitations and Suggestions for Future Research

Based on the social exchange theory, this model offers new theoretical relationships that are needed to be tested empirically. The research opens a call for future studies to test this framework in the field of sales. We limited ourselves to the developmental perception of EPM and focused only on a positive feature. However, the purpose of EPM can also be a deterrent. Earlier research has revealed a negative relationships between deterrent perception of EPM and employees’ outcomes like organizational commitment, felt obligation, job satisfaction, and perceived fairness (Wells et al., 2007). Future research can study the deterrent purpose of EPM’s impact on variables in this study and confirm how it will outline the relationship among variables. The theory of social exchange predicts that if subordinates consider their company is less eager to contribute to social exchange relation, they are also less inclined to engage in social exchange (Blau, 1964). Future studies can examine the implicit employment contract between perceived EPM and ambidextrous behavior of employees. In this study, we could only integrate LMX quality perceived by subordinates. Future researchers can also fill this limitation by including LMX perceived by subordinates and LMX perceived by leaders. Finally, we have proposed this model specifically in the context of the salesforce. However, future research can adapt similar framework in other industries, specifically in banking, where monitoring is the most common and importance of trust and LMX relationship is higher.

## Data Availability Statement

The original contributions presented in the study are included in the article/supplementary material, further inquiries can be directed to the corresponding author.

## Author Contributions

FA and SS initially worked together to finalize the all of parts, including the idea of the work, the conceptual framework, systematic literature review, development of propositions, discussion, and conclusion of the manuscript, and produced the initial draft of the manuscript. FT, YD, and NQ helped in addressing the reviewer’s comments in the manuscript and contributed to re-drafting the manuscript and also re-evaluating the review of the past literature. All authors made substantial contributions to the revision of the manuscript.

## Conflict of Interest

The authors declare that the research was conducted in the absence of any commercial or financial relationships that could be construed as a potential conflict of interest.

## Publisher’s Note

All claims expressed in this article are solely those of the authors and do not necessarily represent those of their affiliated organizations, or those of the publisher, the editors and the reviewers. Any product that may be evaluated in this article, or claim that may be made by its manufacturer, is not guaranteed or endorsed by the publisher.
